# Mini-FLOTAC, a new tool for copromicroscopic diagnosis of common intestinal nematodes in dogs

**DOI:** 10.1186/1756-3305-7-356

**Published:** 2014-08-06

**Authors:** Maria P Maurelli, Laura Rinaldi, Settimia Alfano, Paola Pepe, Gerald C Coles, Giuseppe Cringoli

**Affiliations:** Department of Veterinary Medicine and Animal Productions, University of Naples Federico II, CREMOPAR Regione Campania, Via Della Veterinaria 1, 80137 Naples, Italy; Department of Sperimental Medicine, Second University of Naples, Naples, Italy; School of Veterinary Sciences, University of Bristol, Langford House, Bristol, UK

**Keywords:** Direct smear, Dog, Flotation, Wisconsin, Mini-FLOTAC, FLOTAC, Intestinal nematodes, Sensitivity

## Abstract

**Background:**

The most common intestinal nematodes of dogs are *Toxocara canis*, hookworm and *Trichuris vulpis*. The present study was aimed to validate a new copromicroscopic technique, the Mini-FLOTAC and to compare its diagnostic efficiency and sensitivity with four other copromicroscopic techniques: direct smear, tube flotation, Wisconsin method and the FLOTAC dual technique.

**Findings:**

Two experiments were performed. In the first, faecal positive samples collected from 59 stray asymptomatic dogs, of which 21 were naturally infected with ancylostomidae, 13 naturally infected with *T. canis* and 25 naturally infected with *T. vulpis* were used to validate the Mini-FLOTAC technique. The second experiment was performed on faecal samples randomly selected from 38 stray asymptomatic dogs to compare the diagnostic efficiency and sensitivity of the different techniques. Samples were fixed with 5% formalin; sodium chloride and zinc sulphate were used for flotation solutions because they performed best for detecting and quantifying intestinal nematode eggs in dogs. Mini-FLOTAC and FLOTAC were the most efficient and sensitive techniques and they gave higher EPG and higher numbers of positive samples in both the experiments, for all three parasites.

**Conclusions:**

As Mini-FLOTAC does not require centrifugation it is a very promising technique for counting helminth eggs in dog faeces.

## Findings

### Introduction

The most common intestinal nematodes in dogs are *Toxocara canis*, hookworm and *Trichuris vulpis*[1]. Their control is especially important where there are young children because of their close contact with dogs and in very young children the risk of geophagy, as these parasites are very widespread in environment [2].

The presence of intestinal parasitic infections in dogs is normally checked by copromicroscopic techniques, the most common being direct smear or flotation methods such as the simple tube flotation or the flotation in centrifuge (Wisconsin technique) [3]. The direct smear is a quick technique that requires minimal equipment as well as a small amount of faeces (about 0.1 g). Some veterinarians make direct smears using only the faeces that cling to a rectal thermometer after taking the animal’s temperature. This procedure is inaccurate, with a very low sensitivity, and it also leaves a large amount of faecal debris on the slide, making visualization of eggs more difficult. To overcome these problems flotation techniques have been developed [4].

These are commonly used in parasitological diagnosis because they concentrate parasitic elements and remove debris. The most utilized flotation techniques for canine parasite diagnosis are the flotation in tube and the Wisconsin [5]. It is important to note, however, that these techniques have a low efficiency and may miss low-intensity infections or may be inefficient at high egg density, due to a lack of a grid on the slides, that permit a correct count of parasitic elements inside a known area [6, 7]. Several quantitative microscopic techniques (faecal egg count, FEC) utilizing the flotation method are used in parasitology for the study, diagnosis and counting of parasitic eggs, larvae, oocysts and cysts per gram of faeces (EPG, LPG, OPG, CPG) in animals and humans [8].

The most efficient and sensitive technique for counting eggs, larvae, oocysts and cysts of parasites of dogs is the FLOTAC technique [8], which is accurate to 1 EPG, LPG, OPG, CPG, but requires centrifugation of the apparatus [9]. To simplify the diagnostic procedure, the Mini-FLOTAC apparatus has been introduced and sample preparation has been simplified with the Fill-FLOTAC [10], a sampling kit that eliminates operator contact during processing of samples, i.e. weighing, homogenization, filtration and filling of the Mini-FLOTAC chambers. Likewise to the FLOTAC, Mini-FLOTAC is very useful for multivalent techniques that permit simultaneous diagnosis of eggs, larvae, oocysts and cysts. The present study was aimed at comparing the Mini-FLOTAC technique (MFT) with four other microscopic techniques: direct smear, flotation in tube, flotation in centrifuge (Wisconsin method) and the FLOTAC *dual technique* (FDT). For these purposes, two experiments were performed using faeces samples from dogs naturally infected with *T. canis*, ancylostomidae, and *T. vulpis*.

### Methods

#### Experiment 1

Faecal samples were collected from 59 asymptomatic naturally infected dogs with *T.canis* (13 samples), ancylostomidae (21 samples) and *T.vulpis* (25 samples) that were previously analysed with FDT, using two flotation solutions (FSs), sodium chloride (FS2, specific gravity = 1.20) and zinc sulphate (FS7, specific gravity = 1.35) [9].

Each faecal sample was analysed fresh and fixed in 5% formalin. For all the flotation based techniques the same two FSs previously employed with FDT were used.

From each fresh faecal sample, after a thorough homogenization, one aliquot of 5 g was used to perform 3 replicates of: direct smear (DS) [11], flotation in tube (FT) [3] and Wisconsin (WS) [3]. Then, from each fresh sample one aliquot of 20 g was weighed and fixed with 20 ml of 5% formalin with a dilution ratio of 1:1 (one part of 5% formalin and one part of faeces). After one week, the fixed faeces were diluted with water to reach 400 ml (faecal dilution = 1:20), thoroughly homogenized and filtered through a 250 μm wire mesh. From the filtered suspension, 18 aliquots of 10 ml were placed in 15 ml tubes and then centrifuged for 3 min at 170 × g. The supernatant was discarded and tubes were then randomly assigned to the following techniques: FT, WS, and FDT to have 3 replicates for each of the two FSs used for each technique. For the Mini-FLOTAC technique (MFT) from each fresh faecal sample, two aliquots of 2 g were placed in two Fill-FLOTACs and fixed with 2 ml of 5% formalin. The FS was added to a final volume of 40 ml, homogenized and 3 Mini-FLOTACs were filled for each of two FSs, to have 3 replicates for each FS.

It is important to note that the faecal fixation was performed using 5% formalin in combination with a reduced dilution ratio (1:1) compared to the usual standard used in parasitology (formalin concentration of 10% at the dilution ratio 1:4) [11]. For the sensitivity we have calculated the gold standard based on a combination of all the techniques used.

#### Experiment 2

For the diagnosis of *T.canis*, ancylostomidae and *T.vulpis*, faeces from 38 randomly selected asymptomatic dogs were collected, thoroughly homogenized and used to perform a comparison of different copromicroscopic techniques: DS, FT and WS on fresh faeces, FT, WS, FDT and MFT on fixed faeces. From each fresh faeces one aliquot of 10 g was weighed and fixed with 10 ml of 5% formalin (dilution ratio of 1:1). After one week, the fixed faeces were diluted with water to reach 200 ml (faecal dilution = 1:20), thoroughly homogenized and filtered through a 250 μm wire mesh. From the filtered suspension, 6 aliquots of 10 ml were placed in 15 ml tubes and then centrifuged for 3 min at 170 × g. The tubes were then randomly assigned to the following techniques: FT, WS and FDT for both FSs and when testing the efficiency and sensitivity, no replicates were used.

Two aliquots of 2 g of fresh faeces, from each of the dogs, were placed in two Fill-FLOTACs, fixed with 2 ml of 5% formalin (dilution ratio 1:1) and processed as already described to perform the Mini-FLOTAC.

For the percentage of positive samples, we have calculated the gold standard considering it a combination of all the techniques used.

#### Statistical analysis

The arithmetic mean EPG were calculated for each parasite and each technique. Differences between the EPG obtained with the two FSs were analysed using one-way ANOVA with post hoc Fisher’s least significant difference (LSD). Differences between sensitivity were obtained using chi-square test. All statistical analyses (Mann–Whitney *U*-test, ANOVA) were performed using STATA 10.0 software (Stata Corp., Texas 77845, USA).

### Results

#### Experiment 1

The two FLOTAC techniques (MFT and FDT) gave higher eggs counts for *T. canis* and ancylostomidae (with use of FS2) than DS, FT and WS (*P* < 0.05), whereas for *T. vulpis* FDT with use of FS7 was more sensitive than the other techniques (*P* < 0.05) (Table 1). Comparing results of EPG obtained with fresh and fixed faeces, no statistically significant differences were obtained with FT for all three parasites analysed, while for WS there was a statistically significant increase for all three parasites (Table 1).

**Table 1:** **Mean EPG of** ***T. canis*** **, ancylostomidae,** ***T. vulpis*** **in 59 faecal samples using five copromicroscopic techniques and two flotation solutions, sodium chloride (FS2) and zinc sulphate (FS7)**

Parasite	Fresh faeces (mean EPG)					Faeces fixed in 5% formalin (mean EPG)							
	Direct smear	Flotation in tube		Wisconsin		Flotation in tube		Wisconsin		Mini-FLOTAC		FLOTAC dual technique	
		FS2	FS7	FS2	FS7	FS2	FS7	FS2	FS7	FS2	FS7	FS2	FS7
*T. canis*	0.2^**^	5.8^**^	7.4^**^	20.5^**^	27.0^**^	5.3^**^	6.2^**^	66.6^*^	80.2^*^	118.3	129.3	118.6	134.2
Ancylostomidae	0.1^+^	32.1^**^	17.4^+^	41.9^**^	22.8^+^	29.1^**^	4.9^+^	55.5^*^	30.2^**^	124.8	72.9^*^	92.8	66.8^*^
*T. vulpis*	0.4^+^	4.6^+^	6.2^+^	28.6^+^	36.8^**^	5.6^+^	3.5^+^	43.0^**^	53.1^**^	82.3^*^	73.3^*^	59.5^**^	109.4

Significant differences for different symbols (*,**,^+^) (P < 0.05).

The DS gave the lowest sensitivity, 5.1% for *T.canis*, 1.6% for ancylostomidae and 14.7% for *T.vulpis vs* 100% for all the three parasites for FDT and MFT (Table 2). Comparing results obtained with fresh and fixed faeces for sensitivity, no statistically significant differences were obtained with WS for all three parasites analysed, while for FT for *T.canis* a significant decrease with 5% formalin and FS2 was obtained; for ancylostomidae a significant decrease with 5% formalin and FS7 was obtained, while for *T.vulpis* a significant increase with 5% formalin and FS2 was obtained (Table 2).

**Table 2:** **The sensitivity of the two different preservation methods and of five different copromicroscopic techniques for the diagnosis of** ***T. canis*** **,** ***ancylostomidae*** **and** ***T. vulpis*** **in 59 positive faecal samples**

Parasite	TOT positive samples	Fresh faeces					Faeces fixed in 5% formalin							
		Sensitivity (%)					Sensitivity (%)							
		Direct smear	Flotation in tube		Wisconsin		Flotation in tube		Wisconsin		Mini-FLOTAC		FLOTAC dual technique	
			FS2	FS7	FS2	FS7	FS2	FS7	FS2	FS7	FS2	FS7	FS2	FS7
*T. canis*	13	5.1^**^	66.6^*^	61.5^**^	87.2	92.3	51.3^**^	59.0^**^	94.9	94.9	100	100	100	100
Ancylostomidae	21	1.6^**^	96.8	82.5^*^	100	96.8	98.4	71.4^**^	100	96.8	100	100	100	100
*T. vulpis*	25	14.7^**^	66.6^**^	58.6^**^	89.3	96.0	74.6^*^	65.3^**^	98.6	97.3	100	100	100	100

Significant differences for different symbols (*, **) (P < 0.05).

#### Experiment 2

Nine of the 38 samples (23.7%) of dog faeces were positive, 3 for *T. canis*, 4 for ancylostomidae, 2 for *T. vulpis*. The MFT (with use of FS7) and FDT (with both FSs) gave higher EPG with *T. canis* than DS, FT and WS (*P* < 0.05). Also for ancylostomidae, MFT and FDT using FS2 gave higher EPG than DS, FT and WS (*P* < 0.05), whereas for *T. vulpis* FDT using FS7 was more sensitive than the other techniques (*P* < 0.05) (Table 3).

**Table 3:** **Mean EPG of** ***T. canis*** **, ancylostomidae,** ***T. vulpis*** **in 38 faecal samples using five copromicroscopic techniques and two flotation solutions, sodium chloride (FS2) and zinc sulphate (FS7)**

Parasite	Fresh faeces (mean EPG)					Faeces fixed in 5% formalin (mean EPG)							
	Direct smear	Flotation in tube		Wisconsin		Flotation in tube		Wisconsin		Mini-FLOTAC		FLOTAC dual technique	
		FS2	FS7	FS2	FS7	FS2	FS7	FS2	FS7	FS2	FS7	FS2	FS7
*T. canis*	0^**^	5.5 ^**^	4.5 ^**^	9.5 ^**^	9.5 ^**^	1.5 ^**^	1.5 ^**^	44.5 ^*^	69.5 ^*^	63 ^*^	90	90	102
Ancylostomidae	0 ^+^	29 ^**^	7.3 ^+^	35 ^**^	15 ^+^	42 ^**^	9.3 ^+^	89.3 ^*^	23 ^+^	108	90	120	77.3 ^*^
*T. vulpis*	0.5 ^+^	6.6 ^+^	16.4 ^+^	91.8 ^**^	117.4 ^**^	9.1 ^+^	3.7 ^+^	102.9 ^**^	136.3 ^*^	168 ^*^	194.2 ^*^	135 ^*^	269

Significant differences for different symbols (*, **, ^+^) (P < 0.05).

The DS gave the lowest number of positive samples, 0% for *T.canis*, 0% for ancylostomidae and 50.0% for *T.vulpis* vs 100% for all the three parasites for FDT and MFT (Table 4).

**Table 4:** **The percentage of positive samples of** ***T. canis*** **, ancylostomidae and** ***T. vulpis*** **in 38 positive faecal samples, using five copromicroscopic techniques**

Parasite	TOT positive samples	Fresh faeces					Faeces fixed in 5% formalin							
		Prevalence (%)					Prevalence (%)							
		Direct smear	Flotation in tube		Wisconsin		Flotation in tube		Wisconsin		Mini-FLOTAC		FLOTAC dual technique	
			FS2	FS7	FS2	FS7	FS2	FS7	FS2	FS7	FS2	FS7	FS2	FS7
*T. canis*	3	0	66.7	66.7	66.7	66.7	66.7	66.7	100	66.7	100	100	100	100
Ancylostomidae	4	0	100	100	100	75	100	75	100	100	100	100	100	100
*T. vulpis*	2	50	100	100	100	100	100	50	100	100	100	100	100	100

### Discussion

The availability of a wide range of anthelmintic treatments for dogs has often led to their routine use whether or not the animals are infected. Fortunately there is very little evidence for anthelmintic resistance developing except with hookworms in Australia [12]. Diagnosis before treatment should be a general principle for all parasite infections in all animals, but practitioners either do not undertake faecal egg counts or they usually use the DS [13]. The results of this study suggest that, as previously reported in other papers the DS has a very low sensitivity [13, 14], and so, for faecal egg counting to be widely adopted, a simple sensitive technique is required. The combined use of Fill-FLOTAC and MFT for the first time meets the requirement for a simple sensitive test, not requiring a centrifuge and enables handling and processing of samples with minimum operator exposure as well as permitting the safe use of formalin. With the exception of *T.vulpis* eggs in FS7, MFT was as sensitive as FDT and can therefore be considered the standard for detecting helminth eggs in canine faecal samples. Recent works have shown that MFT is more sensitive than the formol-ether concentration and DS for the diagnosis of soil-transmitted helminths in humans [15–17]. It is also more sensitive than the McMaster for the diagnosis of *Eimeria* in goats [18].

Despite the availability of good canine anthelmintics, high infection rates are still found in many parts of the world indicating lack of systematic diagnosis and treatment [19]. The availability of a simple sensitive technique capable of detecting not only nematode and trematode eggs, but nematode larvae (i.e. *Angiostrongylus*, unpublished observations) and intestinal protozoa, e.g. *Giardia*, means that the recommendations of ESCCAP (European Scientific Counsel of Companion Animal Parasites) http://www.esccap.org for faecal examination before treatment can now be met and health of humans and pets improved.

In conclusion, the present study suggests that the MFT is a promising technique for detecting and counting helminth eggs in dog faeces, and can be used in place of the FDT, in laboratories where the centrifugation step cannot be performed.

## Abbreviations

FEC: Faecal Egg Count
EPG: LPG, OPG, CPG, Eggs, larvae, oocysts and cysts per gram of faeces
MFT: Mini-FLOTAC Technique
FDT: FLOTAC dual technique
FS: Flotation solution
DS: Direct smear
FT: Flotation in tube
WS: Wisconsin.
